# Cross-Sectional and En Face Optical Coherence Tomography of Outer Retinal Morphological Changes Following Retinal Detachment Repair

**DOI:** 10.3390/vision10040074

**Published:** 2026-10-01

**Authors:** Noha A. Abdul Khaliq, Nancy E. Khamis Ahmed, Mohamed A. Kabeel, Mohamed Abd Al-Hakim Zaki, Yousef Fouad, Tamer M. Fathi El Mekkawi

**Affiliations:** Ophthalmology Department, Faculty of Medicine, Ain Shams University, Cairo 11585, Egyptyousef.a.fouad@gmail.com (Y.F.);

**Keywords:** rhegmatogenous retinal detachment, optical coherence tomography, en face OCT, silicone oil, ellipsoid zone

## Abstract

Purpose: To characterize short-term outer retinal changes in silicone oil-filled eyes after primary macula-involving rhegmatogenous retinal detachment (RRD) repair using en face and cross-sectional optical coherence tomography (OCT), and to examine the association of outer retinal features, including foveal ellipsoid zone rosettes (FEZRs), with 12-week postoperative best-corrected visual acuity (BCVA). Methods: This prospective observational study included 35 patients with primary macula-involving RRD who underwent pars plana vitrectomy with silicone oil tamponade with follow-up at 3, 6, and 12 weeks. BCVA, external limiting membrane (ELM)/ellipsoid zone (EZ) integrity, persistent subretinal fluid (PSRF), outer retinal folds (ORFs), and FEZR were evaluated. Results: Mean BCVA improved from 1.96 logMAR at baseline to 0.64 logMAR at 12 weeks, with progressive restoration of foveal ELM/EZ integrity. En face OCT showed high agreement with cross-sectional OCT for PSRF and ORF detection. PSRF was detected in 25.7%, ORFs in 5.7%, and FEZR in 22.9% of eyes; FEZR were more frequent in eyes with poor 12-week visual outcomes. Conclusion: Cross-sectional and en face OCT provided complementary information on early outer retinal remodeling after RRD repair with silicone oil tamponade. Progressive ELM/EZ restoration was associated with better 12-week BCVA, whereas FEZR presence was associated with worse 12-week BCVA. These findings support further prospective evaluation of FEZR as a potential early negative prognostic imaging marker.

## 1. Introduction

Rhegmatogenous retinal detachment (RRD) is a major cause of visual morbidity, with a rising incidence in the recent decades, owing to the growing numbers of pseudophakic patients and refractive surgery procedures [1]. Visual recovery after RRD surgery is influenced by several factors, including macular status, duration to surgery, extent of detachment, persistent subretinal fluid (PSRF), and integrity of the outer retinal layers [2]. Photoreceptor preservation is particularly important, as shorter time to surgery is associated with better photoreceptor recovery and improved visual outcomes [3].

Optical coherence tomography (OCT) has increasingly been used as a non-invasive tool for predicting postoperative outcomes. In contrast, other approaches used to characterize retinal detachment-related changes at the molecular level, such as ionomic profiling of vitreous humor and serum [4], transcriptomic analysis of retinal tissue [5], and vitreous proteomic analysis [6], require invasive sampling of ocular fluid or tissue, underscoring the comparative advantage of OCT as a repeatable, non-invasive imaging modality for longitudinal postoperative monitoring. Outer retinal band integrity on OCT B-scans, including the ellipsoid zone (EZ) and external limiting membrane (ELM), has been correlated with visual recovery after pars plana vitrectomy [7]. En face OCT can complement B-scan imaging by providing a broader assessment of outer retinal status and may help predict visual recovery [8,9]. Additionally, en face OCT can detect outer retinal folds (ORFs) after macula-off RRD repair, where slow fold resolution may correlate with visual gain [10]. It is also useful for detecting PSRF, a factor suggested to delay photoreceptor and visual recovery [11].

The foveal ellipsoid zone rosette (FEZR) was first described as a distinct postoperative pattern of foveal EZ reflectivity after macula-off RRD repair [8]. Although en face outer retinal abnormalities have been followed longitudinally after RRD repair [9], the specific relationship between FEZR and early postoperative BCVA remains insufficiently characterized. Moreover, data describing these changes specifically in silicone oil-filled eyes are limited. Accordingly, the present study evaluated the frequency of FEZR and its association with 12-week BCVA in a prospective cohort of silicone oil-filled eyes.

## 2. Methods

### 2.1. Study Design and Setting

This prospective observational study was conducted on 35 patients who underwent surgical repair of primary RRD by pars plana vitrectomy with silicone oil tamponade at the Ophthalmology Department and Clinics of Ain Shams University Hospitals, Cairo, Egypt, during the period from June 2023 to June 2025. The study was approved by the Research Ethics Committee (REC) of Ain Shams University Faculty of Medicine (approval number: FMASU MD114a/2023). Written informed consent was secured from all participants before enrolment.

### 2.2. Eligibility Criteria

Inclusion criteria were age ≥18 years, primary macula-involving RRD defined as fovea-off or fovea-split detachment, presentation within 6 months from onset of detachment symptoms, and RRD with no PVR or with PVR Grade A or B. Exclusion criteria were chronic RRD presenting beyond 6 months from symptom onset, PVR Grade C or higher, re-detachment or failure of retinal reattachment after a single surgical procedure, exudative or tractional retinal detachment, prior pneumatic retinopexy or scleral buckling, history of glaucoma, uveitis, age-related macular degeneration, retinal vascular occlusion, macular hole, or retinoschisis, and poor-quality OCT scans due to media opacity, poor fixation, or lack of patient cooperation.

### 2.3. Preoperative Assessment

All patients underwent a comprehensive preoperative ophthalmological assessment. BCVA was measured using the Snellen chart and converted to the logarithm of minimum angle of resolution (LogMAR). Ocular tension was assessed using non-contact air-puff tonometry (TOPCON, Japan). Anterior segment examination was performed using slit-lamp biomicroscopy. Dilated fundus examination was carried out using slit-lamp biomicroscopy with a +90 D lens and indirect ophthalmoscopy with a +20 D lens. Clinical documentation included the duration of symptoms before surgery, number of retinal breaks, extent of retinal detachment, and grading of proliferative vitreoretinopathy.

### 2.4. Surgical Technique

All surgeries were performed by the vitreoretinal team at Ain Shams University Hospitals using the Constellation Vitrectomy System (Alcon, Fort Worth, TX, USA) and the RESIGHT wide-angle viewing system (Zeiss, Oberkochen, Germany). Standard three-port 23-gauge transconjunctival pars plana vitrectomy was performed to achieve retinal reattachment and close all retinal breaks. Combined phacoemulsification was reserved for cataractous eyes to improve visualization and facilitate anterior vitreous-base removal. Triamcinolone acetonide was used to visualize residual vitreous and the posterior hyaloid. Traction around retinal breaks was relieved, followed by diathermy marking, air–fluid exchange, and endolaser photocoagulation. Endolaser photocoagulation was applied to the retinal breaks outside the macular area in all cases; no eye received laser treatment within or immediately adjacent to the macula. Silicone oil was used as the endotamponade in all cases. Gas tamponade was not used in this cohort, as sulfur hexafluoride/perfluoropropane gas was not available at our center during the study period; silicone oil was therefore the only endotamponade option.

### 2.5. Postoperative Assessment

Successful retinal reattachment was defined as a flat retina without open retinal breaks or postoperative macular complications, such as macular hole formation or epiretinal proliferation. All patients underwent detailed ophthalmological evaluation at baseline and during follow-up visits at 3, 6, and 12 weeks after surgery. The postoperative assessment included BCVA measurement using the Snellen chart and converted to the logarithm of the minimum angle of resolution (logMAR), ocular tension measurement using non-contact air-puff tonometry (TOPCON, Tokyo, Japan), slit-lamp biomicroscopy for anterior segment examination, and dilated fundus examination using slit-lamp biomicroscopy with a +90 D lens and indirect ophthalmoscopy with a +20 D lens. As IOP was not a predefined primary or secondary study outcome, values were retrospectively retrieved from clinical records where available rather than through a standardized IOP-monitoring protocol. At the final follow-up of 12 weeks, visual outcomes were categorized into poor (≥0.7 logMAR), good (≤0.3 logMAR), or intermediate (0.3–0.7 logMAR).

Imaging was performed using the RTVue XR Avanti spectral-domain OCT (SD-OCT) system (Optovue Inc., Fremont, CA, USA) under mesopic illumination after pupil dilation with 1% tropicamide. High-resolution radial and raster SD-OCT scans centered over the fovea were obtained to evaluate macular microstructure. En face OCT images were generated from the structural mode of the HD Angio Retina 6 × 6 mm scan using reflectivity-based segmentation of customized outer-retinal slabs. Slab boundaries were selected according to the structure being evaluated, extending from the external limiting membrane to immediately above the retinal pigment epithelium for PSRF assessment and from the outer plexiform–outer nuclear layer junction to the EZ for RF assessment, using the Optovue AngioVue software. Since no standardized description exists for retinal detachment-related changes on en face OCT, all findings were validated using corresponding OCT B-scans through spatial and morphological correlation. All cross-sectional and en face OCT scans were independently evaluated by two graders (A.F.A. and M.A.Z.), masked to BCVA and clinical outcomes. Cross-sectional OCT B-scans served as the reference standard for PSRF and ORF detection, with en face OCT assessed as a complementary imaging method. Disagreements were adjudicated by a third masked senior retinal consultant.

### 2.6. Evaluation of Outer Retinal Morphological Features

Qualitative spectral-domain OCT assessment of the ELM and EZ focused on their continuity, reflectivity, and structural integrity. Disruption was defined as attenuation, fragmentation, or loss of the hyperreflective signal, and ELM/EZ integrity was graded according to the classification proposed by Hänsli et al. [7] from Grade 1 (intact) to Grade 4 (completely absent).

PSRF was identified on SD-OCT B-scans as a well-demarcated hyporeflective space between the neurosensory retina and the retinal pigment epithelium (RPE). Based on morphology, PSRF was classified into bleb-like, shallow-diffuse, and multiple bleb subtypes. Morphological characteristics, foveal involvement, and interval changes, including resolution or persistence, were qualitatively assessed during follow-up and correlated with postoperative visual outcomes. En face evaluation of PSRF was performed using structural en face OCT images generated from a standardized 6 × 6 mm macula-centered OCTA acquisition. The outer-retinal slab extended from the ELM to immediately above the RPE.

ORFs were identified on SD-OCT B-scans as hyperreflective vertical or rounded lesions involving the outer retinal layers and extending into the outer nuclear layer. Folds were considered significant when visible on at least two adjacent raster scans, and their location relative to the fovea was documented. Structural findings were correlated with en face OCT reconstructions and postoperative visual outcomes. En face OCT evaluation was performed using customized slabs extending from the outer plexiform–outer nuclear layer junction to the EZ.

FEZRs were defined as a central hyperreflective focus with radiating striations on en face OCT as previously described [8].

### 2.7. Statistical Methods

Data were analyzed using IBM SPSS Statistics version 27.0 (IBM Corp., Armonk, NY, USA). Quantitative data were presented as mean ± standard deviation or median and interquartile range according to data distribution, while qualitative data were expressed as frequencies and percentages. Normality was assessed using the Kolmogorov–Smirnov test. Statistical tests were selected according to variable type and distribution and included the Chi-square test, Fisher’s exact test, independent-samples *t*-test, Mann–Whitney U test, Wilcoxon signed-rank test, one-way ANOVA, Kruskal–Wallis test, repeated-measures ANOVA, Friedman test, and Spearman correlation analysis. For the diagnostic accuracy component, cross-tabulated 2 × 2 results were used to calculate sensitivity, specificity, and overall accuracy, with 95% confidence intervals estimated using the exact binomial (Clopper–Pearson) method. Receiver operating characteristic (ROC) curves and area under the curve (AUC) values were retained as descriptive secondary measures. A *p*-value < 0.05 was considered statistically significant.

For analyses involving small subgroups or sparse events, Fisher’s exact test was used when expected cell frequencies were ≤5. Subgroup analyses involving fewer than 10 eyes were considered exploratory. No multivariable adjustment or formal correction for multiple comparisons was performed; therefore, secondary association analyses were interpreted cautiously. This study is reported in accordance with the STROBE guideline for observational studies and, for the diagnostic accuracy component (en face OCT versus cross-sectional OCT as reference standard), the STARD guideline for diagnostic accuracy studies.

## 3. Results

Thirty-five eyes of 35 patients were included in the final analysis. Baseline demographic and clinical characteristics are summarized in Table 1. Median age was 47 years (IQR, 39–57), and 60.0% of patients were male. The median duration of symptoms was 7 days (IQR, 2–14).

BCVA improved significantly following surgery, with mean BCVA improving from 1.96 logMAR at baseline to 0.64 logMAR at 12 weeks (*p* < 0.001). Patients with good 12-week visual outcomes had better baseline BCVA (1.18 ± 0.16 logMAR) compared with those who achieved intermediate (2.03 ± 0.47 logMAR) or poor outcomes (2.28 ± 0.18 logMAR; *p* = 0.004).

Postoperative IOP measurements, although not part of a predefined monitoring protocol, were available for 27 of the 35 eyes (77.1%) at one or more follow-up visits (23/27 eyes at 3 weeks, 18/27 at 6 weeks, and 18/27 at 12 weeks). Among these eyes, IOP values ranged from 7 to 30 mmHg (mean 14.4 mmHg). Four eyes (14.8% of those with available data) recorded an IOP > 21 mmHg on at least one occasion; two of these four required the addition of topical brimonidine, and none required surgical IOP-lowering intervention.

**Figure 1:**
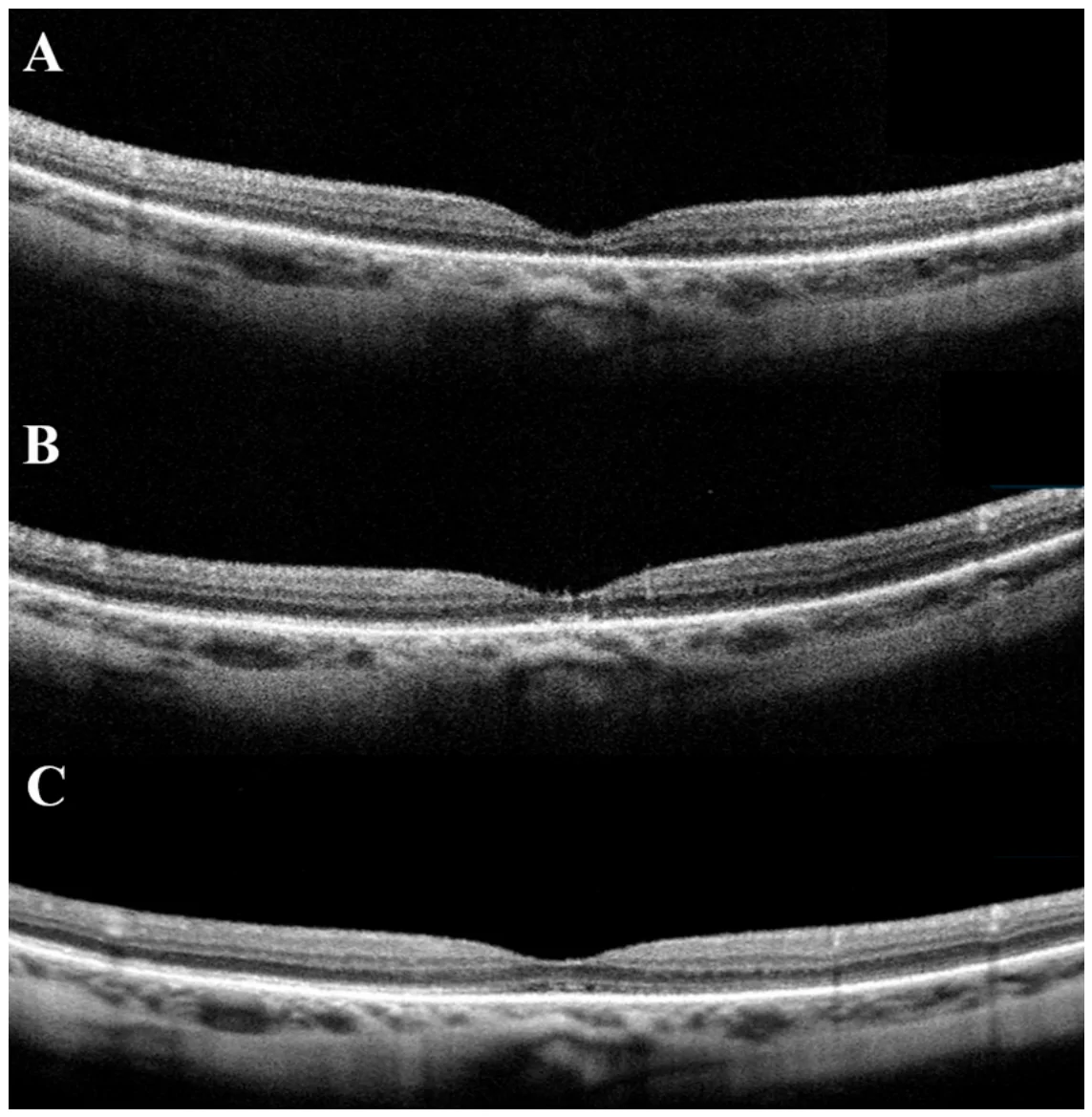
Sequential OCT images showing photoreceptor recovery after retinal detachment repair: (**A**) at 3 weeks, the ellipsoid zone (EZ) is entirely absent (Grade 4); (**B**) at 6 weeks, the ELM and EZ show discontinuity and fragmentation (Grade 3); (**C**) by 12 weeks, the ELM is intact while the EZ has improved to Grade 2 (altered but continuous) Arrows are omitted for clarity; grading follows the ELM/EZ classification of Hänsli et al. [7], with Grade 1 representing fully intact bands and Grade 4 representing complete absence of both ELM and EZ signal.

OCT analysis demonstrated progressive recovery of the ELM/EZ bands, with a significant shift from absent to intact integrity by 12 weeks (Figure 1). The 12-week BCVA was strongly associated with foveal photoreceptor integrity. By 12 weeks, intact foveal layers were observed only in eyes achieving excellent visual outcomes (<0.3 logMAR) compared to those with poorer visual outcomes (logMAR VA > 0.3, *p* < 0.001).

**Figure 2:**
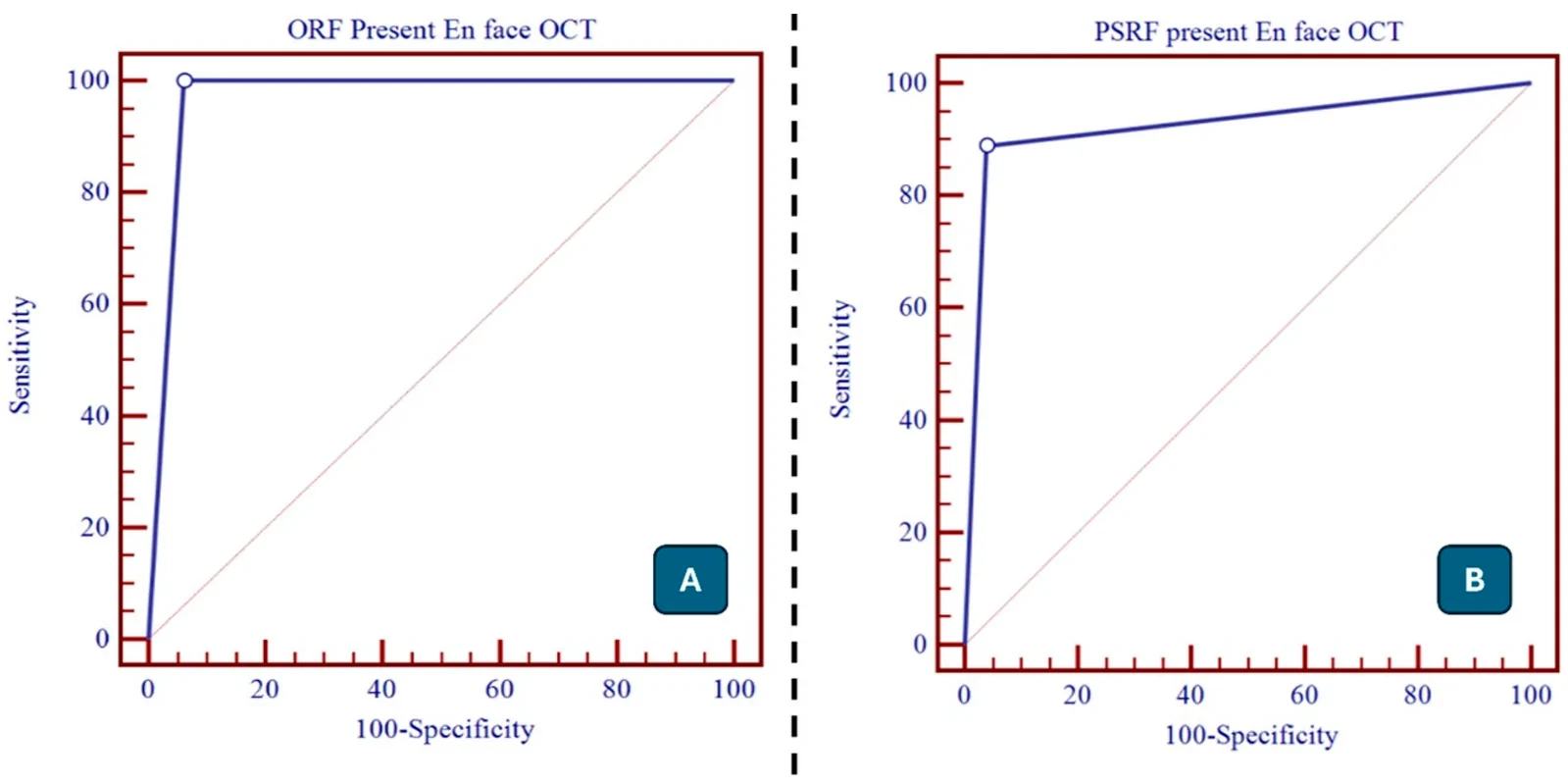
Receiver operating characteristic curves for en face OCT detection of (**A**) outer retinal folds (ORF), and (**B**) persistent subretinal fluid (PSRF). Cross-sectional OCT B-scans served as the reference standard in both analyses; area under the curve (AUC) values were 0.89 for ORF and 0.94 for PSRF, with corresponding sensitivity, specificity, and accuracy reported in Section 3.

**Figure 3:**
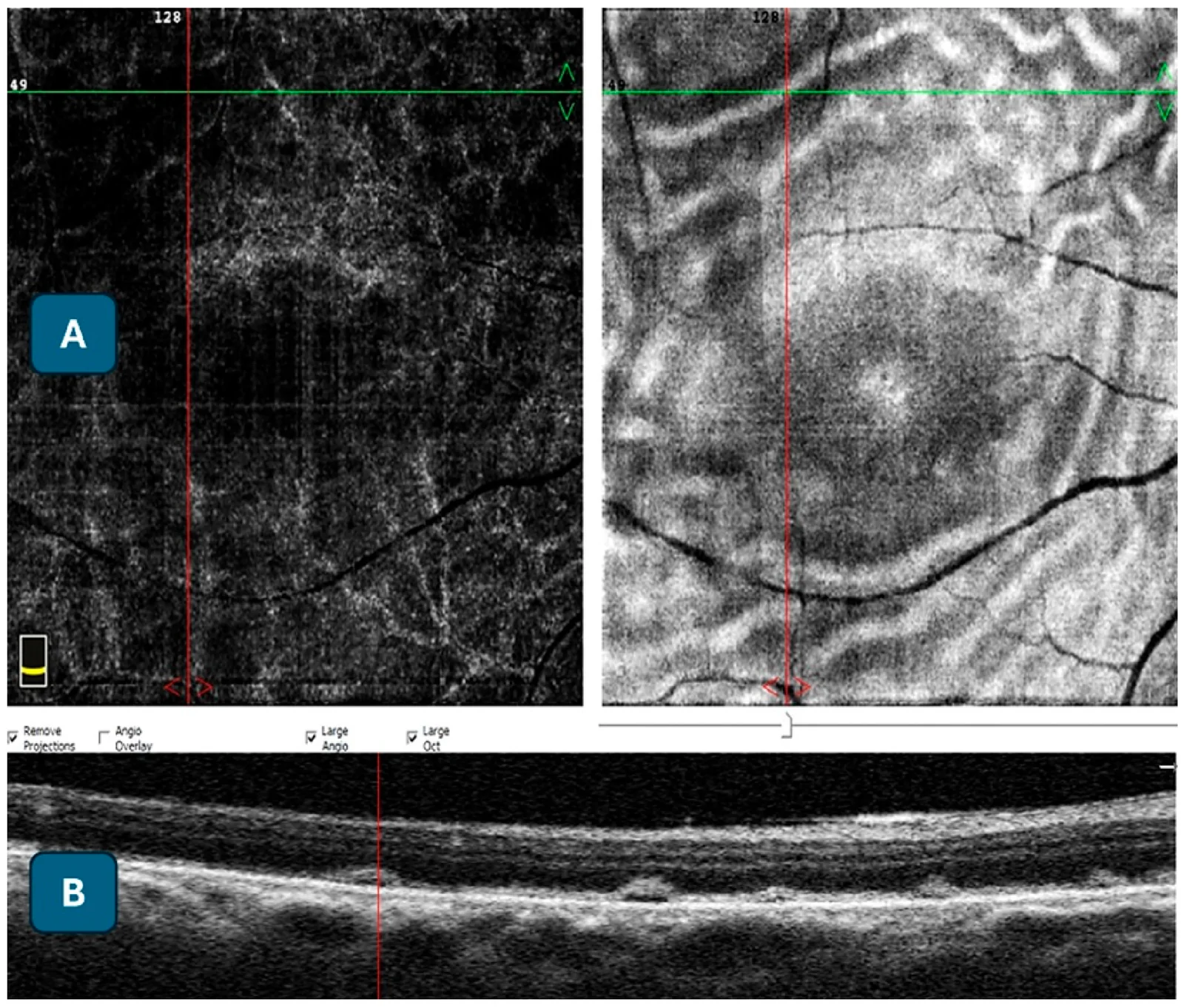
False-positive en face outer retinal folds appearance: (**A**) en face OCT shows linear and curved hyperreflective lines suggestive of outer retinal folds; (**B**) the corresponding cross-sectional OCT B-scan demonstrates PSRF blebs without a true outer retinal fold, illustrating a source of potential overcalling of ORF on en face imaging alone and the rationale for using cross-sectional OCT as the reference standard.

En face OCT showed high agreement with cross-sectional OCT for PSRF detection, with a sensitivity of 88.9% (95% CI, 51.8–99.7%), specificity of 96.2% (95% CI, 80.4–99.9%), and overall accuracy of 94.3% (95% CI, 80.8–99.3%); the AUC was 0.94. For ORF detection, sensitivity was 100% (95% CI, 15.8–100.0%), specificity was 93.9% (95% CI, 79.8–99.3%), and accuracy was 94.3% (95% CI, 80.8–99.3%); the AUC was 0.89 (Figure 2). The wide confidence interval around ORF sensitivity reflects that ORFs were identified in only two eyes; these estimates should therefore be considered preliminary. An example of false-positive assessment of ORFs is demonstrated in Figure 3.

**Figure 4:**
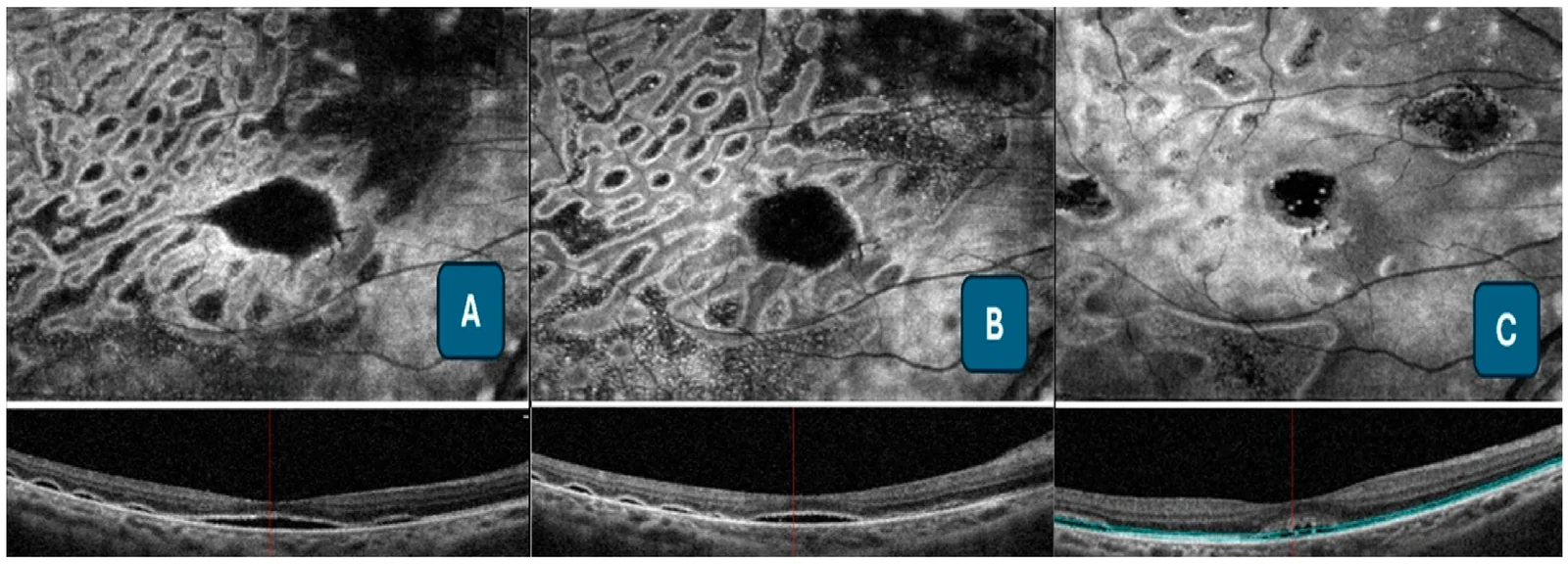
Sequential en face OCT images showing reduction in persistent subretinal fluid (PSRF) over 12 weeks: (**A**) large multilobular hyporeflective PSRF at 3 weeks; (**B**) noticeable reduction at 6 weeks; (**C**) further decrease with small residual pockets at 12 weeks. Corresponding cross-sectional OCT B-scans confirmed the en face findings at each time point through spatial and morphological correlation.

PSRF was detected in nine eyes (25.7%) following retinal detachment repair. Among the affected cases, the most frequent pattern was multiple blebs (66.7%). Foveal involvement was present in 33.3% of PSRF cases. Regarding the natural course, PSRF showed a tendency toward improvement in most cases, with 22.2% resolving completely and 44.4% demonstrating partial reduction (Figure 4), while 33.3% persisted throughout the 12-week follow-up. Importantly, no cases exhibited progression or increase in fluid. Among PSRF-positive eyes, foveal involvement was associated with reduced BCVA improvement from baseline to 12 weeks (BCVA gain, *p* = 0.014), suggesting delayed early visual recovery. However, PSRF presence, morphology, and evolution were not significantly associated with 12-week BCVA (all *p* > 0.05).

ORFs were identified in two of 35 eyes (5.7%) and were paracentral in both cases, with no foveal involvement. Both ORF-positive eyes showed a reduction in fold prominence during follow-up, with no progression or complete resolution within the study period. ORF presence, location, and evolution were not significantly associated with 12-week BCVA.

**Figure 5:**
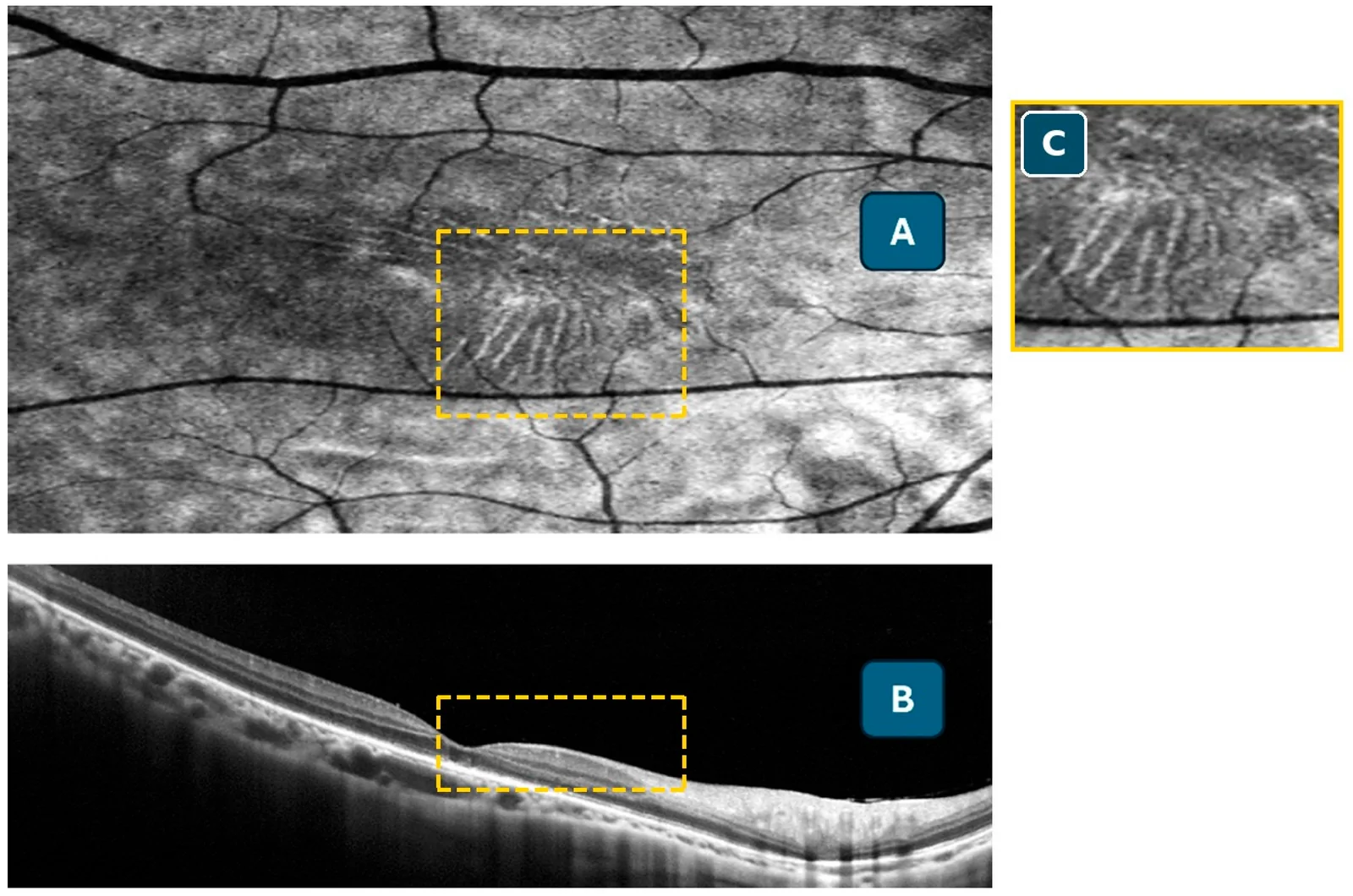
Foveal ellipsoid zone rosette (FEZR): (**A**) structural en face OCT demonstrates a central hyperreflective focus with radiating striae and intervening hyporeflective clefts; (**B**) the corresponding cross-sectional OCT shows focal EZ disruption adjacent to preserved EZ; (**C**) magnified inset of the boxed region in (**A**), highlighting the radiating striae and hyporeflective clefts of the rosette. Dashed boxes in (**A**,**B**) denote the corresponding FEZR region on en face and cross-sectional imaging, respectively.

FEZR were identified in 22.9% of eyes (Figure 5). FEZR presence was significantly associated with poorer 12-week BCVA (*p* = 0.008), being absent in all good visual outcome eyes (logMAR < 0.3) and present in 62.5% of poor visual outcome eyes.

## 4. Discussion

The present study highlights the value of SD-OCT B-scans and en face OCT as complementary methods in the assessment of outer retinal morphological features following successful repair of RRD that may serve as predictors of visual outcomes.

Significant BCVA improvement was noted at 12 weeks postoperatively. Baseline BCVA was significantly associated with 12-week visual outcome, consistent with the prior literature [2]. Final BCVA was significantly associated with foveal status at presentation, consistent with the prior literature [12], whereas the number of retinal breaks, extent of detachment, PVR grade, and duration of symptoms were not significantly associated with 12-week visual outcome. The lack of a significant association with symptom duration in our cohort may be related to the relatively short and limited distribution of symptom duration, with a median of 7 days and a maximum of 30 days, resulting in relatively few cases with prolonged detachment and potentially limiting the ability to detect a duration-dependent effect on visual recovery. Nonetheless, within this range, eyes operated on earlier after symptom onset did not show a consistently different postoperative outer retinal or visual recovery pattern from those operated on later, arguing against a strong duration effect specifically within the 1–30-day window studied here; a wider range of presentation intervals would be needed to fully evaluate this factor.

Cross-sectional OCT showed progressive restoration of photoreceptor integrity after RRD repair, with a significant shift from absent or interrupted layers toward altered or intact ELM/EZ integrity by 12 weeks. Better foveal photoreceptor integrity was strongly associated with better 12-week BCVA. These findings are consistent with Kobayashi et al. [13], who reported that recovery of the EZ and the presence of a foveal bulge were associated with better visual recovery. The findings are also in line with those reported by Hänsli et al. [7], who found that the integrity of the ELM, EZ, and interdigitation zone on OCT B-scans correlated with postoperative visual outcomes after RRD repair.

En face OCT demonstrated high agreement with cross-sectional OCT in detecting PSRF and facilitated visualization of its extent and distribution across the macula. Similarly, high diagnostic indices were observed for ORF detection, although the relatively low prevalence of ORFs in this cohort limits definitive conclusions regarding diagnostic accuracy. These findings highlight the complementary role that en face OCT lends to cross-sectional OCT B-scans in postoperative retinal assessment and support their integration together rather than using a standalone imaging modality.

PSRF was detected in 25.7% of eyes, with multiple blebs being the most frequent morphology. Most cases showed either complete resolution or partial reduction during follow-up, while no case showed progression. These findings are consistent with a previous study by Kim et al., who reported multiple SRF blebs as the major presentation of PSRF and described their natural history as gradual reduction over time [14]. The incidence in the present study is higher than the 3–19% range summarized by Fouad et al. [11], which may be related to the early timing of postoperative assessment and the use of en face OCT.

Regarding visual outcome, PSRF morphology, PSRF foveal involvement, and evolution were not significantly associated with 12-week BCVA. Conflicting results exist in the prior literature regarding the association between PSRF and BCVA improvement and whether such association is subtype dependent. Fu et al. [15] found poorer early VA in eyes with PSRF, although this difference disappeared after longer follow-up. Benson et al. [16] found no significant difference in final BCVA in eyes with different PSRF patterns, but Ricker et al. [17] reported worse postoperative VA with multiple blebs and shallow-diffuse PSRF compared to those with a single bleb pattern. These differences may reflect variations in follow-up duration, PSRF morphology, and timing of OCT evaluation.

ORFs were uncommon in our cohorts, detected in only 5.7% of eyes, and all were paracentral without foveal involvement. Both eyes with folds showed reductions in fold prominence over time, and no significant association was found with 12-week BCVA. Our findings differ from prior studies that report higher ORF rates [18,19,20] but are consistent with them in the natural history of the folds declining over time, suggesting spontaneous improvement. The lower incidence in the present study may be related to the surgical technique, tamponade agent used and postoperative positioning. Although ORFs were not related to 12-week BCVA in the present study, metamorphopsia was not assessed. This is important because Fukuyama et al. [21] showed that fold density measured on en face OCT correlated with metamorphopsia using M-CHARTS. Therefore, the absence of a BCVA association does not exclude a possible effect of ORFs on visual quality or distortion.

FEZR was detected in 22.9% of eyes and was significantly associated with poorer 12-week BCVA. FEZR was absent in all eyes with very good visual outcomes and was most frequent among eyes with poor vision. Thomseth et al. [8] described FEZR as a characteristic foveal imaging finding after RRD repair, appearing as a hyperreflective foveal rosette on multimodal imaging and corresponding to localized EZ disruption on OCT. In the present cohort, FEZR should therefore be interpreted as a structural feature associated with poorer concurrent 12-week visual function rather than as a validated prognostic marker. Its potential predictive value requires confirmation in larger studies in which FEZR is assessed before a later visual outcome. The broader pattern of progressive EZ restoration on en face imaging parallels the longitudinal recovery trajectory described by Figueiredo et al. [9]. We did not formally test preoperative factors (e.g., duration of symptoms, extent of detachment, or PVR grade) as predictors of FEZR occurrence, as this was beyond the scope of the present analysis. Regarding its pathogenesis, FEZR is thought to arise from localized misalignment and clumping of regenerating photoreceptor outer segments at the fovea during the reattachment process, producing the radiating, striated hyperreflective pattern on en face imaging together with focal EZ disruption on cross-sectional OCT [9]; irregular apposition between recovering photoreceptors and the retinal pigment epithelium at the site of the original detachment may further contribute to this focal disorganization, which could explain its association with poorer concurrent visual function.

This single-center study is limited by its small sample size and 12-week follow-up. ORFs were detected in only two eyes, so the diagnostic accuracy estimates for this finding should be considered exploratory. BCVA was measured with silicone oil still in situ, and lens changes in phakic eyes may have contributed to the patients’ visual performance. Furthermore, visual function was assessed by BCVA alone, without metamorphopsia or other quality-of-vision measures. Multivariate regression analysis for predictors of visual improvement was not possible owing to the small sample size. Postoperative IOP was not part of a predefined monitoring protocol and was therefore unavailable for eight of the 35 eyes and incomplete across visits for others; although the available measurements suggest a low rate of IOP elevation that was managed medically with topical therapy alone, we cannot exclude an effect of unrecorded or transient IOP elevation on the outer retinal findings, particularly in eyes without available data.

In conclusion, following primary macula-involving RRD repair with silicone oil tamponade, foveal ELM/EZ integrity progressively improved during the first 12 weeks after surgery, and this is associated with BCVA recovery. En face OCT provides complementary visualization of PSRF, ORFs, and FEZR alongside cross-sectional OCT. PSRF and ORFs were generally non-progressive and did not alter visual outcomes during follow-up, whereas FEZR presence at 12 weeks was associated with poor BCVA.

## Figures and Tables

**Table 1:** Baseline demographic and clinical characteristics of the studied patients.

Characteristic	Category/Summary	n (%) or Value
Age, years	Median (IQR); range	47 (39–57); 20–65
Sex	Male; Female	21 (60.0%); 14 (40.0%)
Affected eye	Right; Left	11 (31.4%); 24 (68.6%)
Duration of symptoms, days	Median (IQR); range	7 (2–14); 1–30
Lens status	Phakic; Pseudophakic	24 (68.6%); 11 (31.4%)
Foveal status	Fovea-off; Fovea-split	30 (85.7%); 5 (14.3%)
PVR grade	None; A; B	9 (25.7%); 20 (57.1%); 6 (17.1%)
Number of retinal breaks	1; 2; 3; 4 breaks	17 (48.6%); 13 (37.1%); 4 (11.4%); 1 (2.9%)
Extent of detachment	1; 2; 3; 4 quadrants	2 (5.7%); 19 (54.3%); 13 (37.1%); 1 (2.9%)

## Data Availability

The original contributions presented in this study are included in the article. Further inquiries can be directed to the corresponding author.
